# Identification and validation of immunogenic potential of India specific HPV-16 variant constructs: *In-silico* & *in-vivo* insight to vaccine development

**DOI:** 10.1038/srep15751

**Published:** 2015-10-28

**Authors:** Anoop Kumar, Showket Hussain, Gagan Sharma, Ravi Mehrotra, Lutz Gissmann, Bhudev C. Das, Mausumi Bharadwaj

**Affiliations:** 1 Division of Molecular Genetics & Biochemistry; Noida, Uttar Pradesh, India; 2 Division of Cytopathology; Institute of Cytology & Preventive Oncology (ICMR), Noida, Uttar Pradesh, India; 3 Division of Genome Modification and Carcinogenesis, German Cancer Center, DKFZ Heidelberg, Germany; 4 Dr. B.R. Ambedkar center for Biomedical Research, University of Delhi (North Campus), New Delhi, India

## Abstract

Cervical cancer is one of the most common gynecological cancers in the world but in India, it is the top most cancer among women. Persistent infection with high-risk human papillomaviruses (HR-HPVs) is the most important risk factor. The sequence variation(s) in the most common HR-HPV *i.e.* HPV type 16 leads to altered biological functions with possible clinical significance in the different geographical locations. Sixteen major variants (V1-V16) in full length L1 gene of HPV-16 were identified following analysis of 250 prospectively collected cervical cancer tissue biopsies and their effect on immunogenicity was studied. The effect of these major variations on the epitopes were predicted by *in silico* methods and the immunogenicity of variants and respective reference DNA vaccine constructs were evaluated by administration of prepared DNA vaccine constructs in female BALB/c mice to evaluate antibody titer. In the present study, L500F (V16) variation showed a significant ~2.7 fold (p < 0.002) increase in antibody titer, whereas T379P (V8) showed ~0.4 fold (p < 0.328) decrease after final injection. These results showed a promising roadmap for the development of DNA based vaccine and for the generation of effective response, though there is a need to study more prevalent variants of HPV in the Indian population.

Cervical cancer (CaCx) is the third most common cancer among women worldwide with an estimate of 527,624 new cases diagnosed annually and is the most common gynecological cancer in developing counties like India^1^. Several studies showed that persistent infection with high risk Human Papillomavirus (HR-HPV) is an etiological factor for development of CaCx and HPV type 16 and 18 are associated with >70% of cases worldwide^2^. The incidence of CaCx in different countries are associated with distribution of specific viral variants in E6, E7, L1, L2 and long control regions (LCR)^3^ and on the basis of the sequence analysis, the sequence difference by 2% were classified as viral variants^4^. HPV-16 is an ~8 kb dsDNA virus belonging to family papillomaviridae and genus Alphapapillomavirus^5^. HPV-16 has been divided into five different phylogenic lineages-European(E), Asian(As), Asian-American(AA), African(Af) and North-American(NA)^6^. In India, HPV-16 alone contribute to >90% of the cancer of uterine cervix^7^,^8^,^9^. This could be due to HPV intratype variants, which may have different biological and pathological consequences with respect to disease progression^10^.

Identification of HPV as a major causative agent for cervical cancer gives an opportunity to prevent it by vaccine development. The major capsid (L1) and minor capsid (L2) proteins of HPV are attractive candidates and are extensively used for prophylactic vaccine development as they induce virus-specific immune response and have highly immunogenic repetitive epitopes on the surface of virions and have no oncogenic activity. Earlier studies have reported that variations in L1 gene can affect the viral assembly, immunological recognition by the host and immortalization activity which ultimately affect the protein structure or conformation and lead to altered biological functions with clinical significance^11^,^12^.

The role of intra-type variants among HPVs cannot be ruled out; therefore, intratype genomic diversity of HPV sequence is important for the development of efficient diagnostic/prognostic tools and vaccine development. For efficient vaccine, the recognition of correct epitope sequence is important for the generation of efficient immune response^13^. The immunological reaction is important to identify antigen/epitopes and their interaction with major histocompatibility complex alleles for inducing effective B-and T-cell responses for effective vaccine development^13^,^14^. Epitopes derived from reference/prototype may undergo some variation in amino acid located in epitopes critical for the immune response against the pathogen. Alteration in one or more amino acid within the L1 protein of HPV-16 could represent a conformational change in the protein and thus could also affect the conformation of epitopes relevant for viral neutralization^15^.

It is, therefore, imperative to understand the geographical variants of HPV for better targeting the vaccines against it. In India, very limited studies have been carried out on molecular variant analysis of full length L1 of HPV-16^16^,^17^,^18^. The previous studies have reported mainly the variations in L1, the major capsid protein of HPV-16 genome, whereas the present study reports here the effect of Indian major variants of L1 on the epitope change (*in-silico)* as well as on potential immunogenicity *in-vivo* (BALB/c mice).

## Results

### Prevalence of HPV infection

Out of 250 tumor biopsies, 231 showed HPV infection (92.4%) of which 221/231 (95.6%) samples harbored HPV-16; 4/231 (1.7%) was infected with HPV-18, 2/231 (0.8%) showed co-infection of both HPV-16/HPV-18 and the remaining 4/231 (1.7%) had infection with other HPV sub types.

### Variant analysis

We observed 16 major variations (V1-V16) in full length L1 (Table 1); 13 biallelic variations, one trialleic [G7058A/T(V16)] and two frameshift variations; one insertion [ATC insertion at C6901(V12)] and one deletion [deletion of GAT 6590(V13)]. In 13 biallaelic variations, six variations C6163A(V1), G6171A(V2), C6240G(V3), A6432G(V6), G6693A(V8) and C6863T(V11) were missense and seven variations T6245C(V4), A6314G(V5), C6557T(V7), G6719A(V9), C6852T(V10), C6968T(V14) and A6293C(V15) were silent.

On further analysis, it was observed that variations V3, V12 and V13 were observed in all HPV 16 positive samples (100%), which correspond to amino acid change from histidine to aspartate at position H228D, an insertion of serine residue at 448 and deletion of aspartate residue at 465 position respectively (Table 1). V6 corresponding to change in amino acid at T292A was found in ~97% of the samples. Variations V1, V2, V8 and V11 led to change in amino acid at T202N, A205T, T292A, T379P, P435L, respectively and was found in ~25% of the same samples. Besides these, other seven variations (V4, V5, V7, V9, V10, V14 & V15) were found in ~25% of the samples except variation V10 which was observed in ~35% of the samples. V16 variation was triallelic and observed in ~36% of the samples. It causes a change from G to T corresponding to a change in L500F amino acid in ~25% of the samples. However, G to A nucleotide change at the same position was found in ~11% samples which did not correspond to any change in the amino acid.

### Analysis of Structure and Epitope Prediction

**Figure 1:**
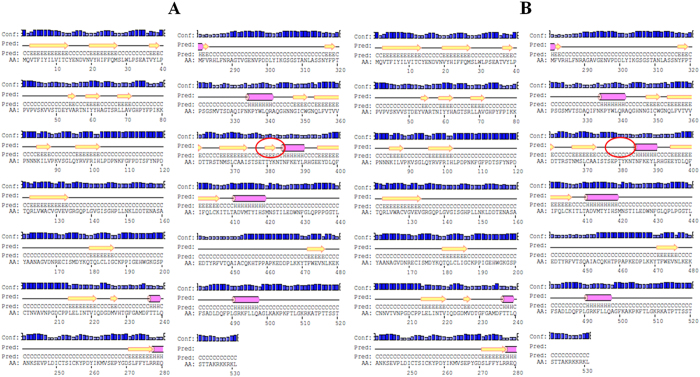
PSIPRED graphical results from secondary structure prediction of L1 gene ORF, (**A**) Reference Sequence; (**B**) Variant Sequence (Change shown in circle).

**Figure 2:**
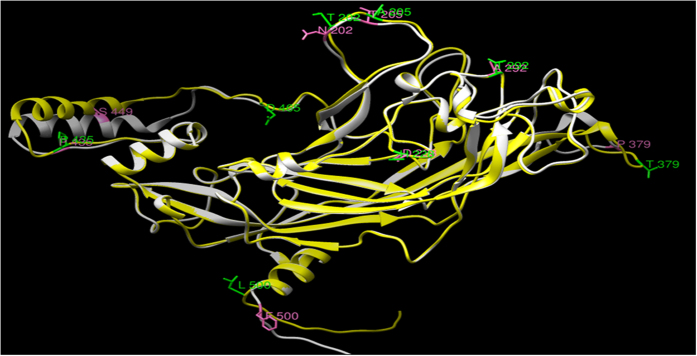
Major Indian variants of full length L1 on the superimposed 3D modeled structure of the reference and variant protein (PDB ID: 1DZL taken as template for the modeling of protein).

Amino acid composition of major capsid protein was compared for both reference and variant, which showed that threonine (T), leucine (L) and proline (P) were the most prominent amino acids with threonine being the most variable amino acid noted. There is an increase in frequency of asparagine, phenyl alanine and serine while there is a decrease in frequency of histidine, threonine in the mutant as compared to reference (Supplementary Table S1). The present study also demonstrated alteration of the hydrophobicity of amino acid residues when compared to hydrophobicity index of each amino acid caused by the variations (Table 1). The secondary structure showed that the reference sequence consisted of 70% (372) coiled (C), 6.8% (36%) helix (H) and 23.2% (123) sheets (E) and the variant sequence consisted of 70.8% (376) coiled, 6.8% (36%) helix and 22.4% (119) sheets. The *in-silico* analysis showed the replacement of threonine by proline at 379 causing distortion of a sheet structure (disappeared), that may be due to the unusual structure of proline (Fig. 1). Figure 2 shows the superimposed variant and reference 3D structure with marked change in amino acid due to variations. A refined alignment of the template (1DZL) and the protein sequence was performed using the Align2D script of modeller program, which considers the structure information of the template in alignment construction. Using this alignment as input, ten structural models were generated. The structure fulfilling all the structural constraints was chosen in accordance with the Ramachandran plot of the 3D-model (Supplementary Fig. S1). Ramachandran plot of 3D structures is the general analysis method for determining the overall structure equivalence of model with that of known structures. Both the modeled reference and variant protein contained 88.4% residues in the core region of plot, while there were 11.4% and 11.0% residues in the allowed region of reference and variant and a less than 0.7% residues comes under the generously allowed and disallowed region of the modeled proteins. Furthermore, the stereo-chemical property of 531 amino acids model structure was verified using the Structural Analysis and Verification Server (SAVES). PROCHECK program was used to check the stereo-chemical excellence and the overall structural geometry of the homology model. VERIFY3D program was used to determine the compatibility of the atomic model (3D) with its own amino acid sequence (1D) by assigning a structural class on the basis of its location and environment (alpha, beta, loop, polar, non-polar, etc.) as well as comparing the results with good database structures. Many stereo-chemical parameters of the residues in the model were ensured for their authenticity by WHATCHECK program.

We identified India specific major variations of L1, which may play an important role in immunogenicity. Previously, it was showed that amino acids from 494 to 518 of L1 were known as hypervariable epitope constructs (HEC) regions^19^. HECs showed the broad immune reactivity to related epitope analogues capable of overcoming immunogenic peptide for different strains and inducing antibodies against them. In addition, HEC regions corresponding to 289–308 and 494–518 of amino acids on the L1 capsid protein of HPV were known to have B-cell epitopes^19^,^20^. Therefore, we prepared DNA vaccine construct for L500F (V16) variation, which was found in the vicinity of HEC regions amino acid 469–493 on the L1 capsid protein of HPV. We also predicted epitopes for other variations and prepared their construct for evaluation of their effect on the immunogenicity (Data not shown). Therefore, in addition to V16, we also prepared a DNA vaccine construct for V8 and the predicted epitope in the reference sequence ISTSETTYKNTN had a score of 0.636 (with Thr having score 0.833) and in variant sequence, the predicted epitope ISTSEPTYKNT had a score of 0.630 (with Pro having score 0.796). These results showed that replacement of threonine by proline reduced the immunogenicity which may be due to the structural constraint caused by the unusual shape of proline.

**Figure 3:**
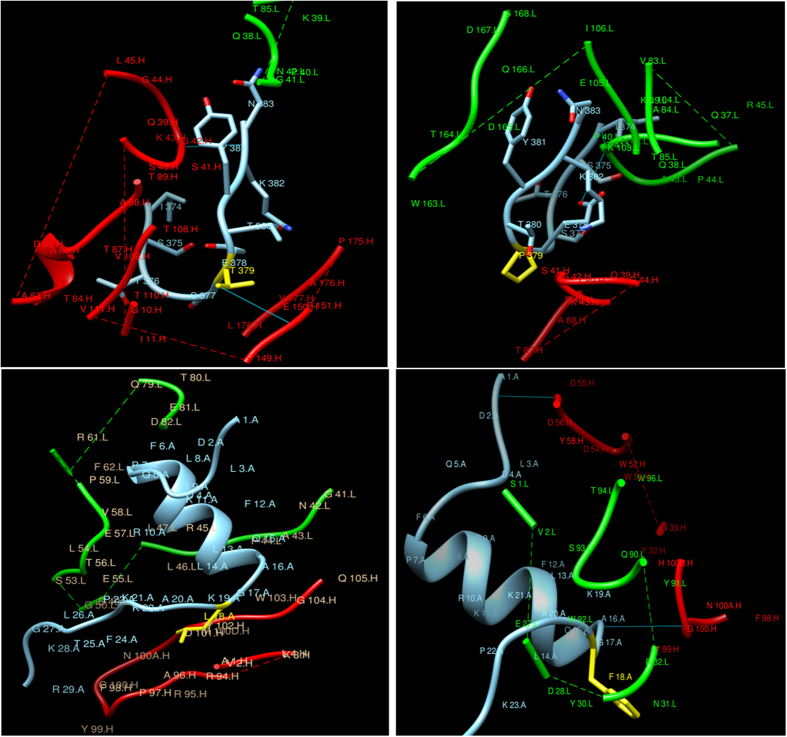
Docking of epitopes of respective reference (8R and 16R) and variant (8V and 16V) with antibody (PDB ID: IJRH). Arrows show the change in interaction due to variations.

Structure of the epitope was predicted and docked with antibody (1JRH) using Patchdock and best model refinement was done by Firedock. The best docked model having lowest global energy for V16R, V16V, 8R and 8V were selected and visualized in chimera. The comparison of respective reference and the variant peptide showed a new hydrogen bond in case of V16V and loss of hydrogen bond in case of V8V, which also causes change in global energy i.e. −61.67 for V8R and −54.60 for V8V, where as in for V16R and V16V were −49.55 and 50.86, respectively (Fig. 3). In V8V, the replacement of threonine (T) by proline (P) caused the loss of hydrogen bond. These results also indicated the change in binding affinity due to these variants.

### Evaluation of Immunogenicity of HPV-16 variant constructs in animal model

**Figure 4:**
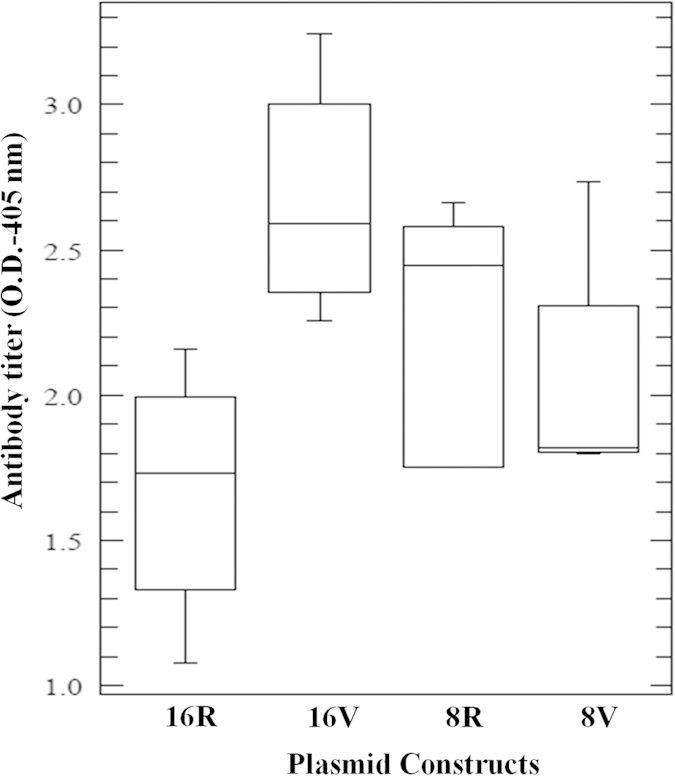
Serum IgG antibody response by box-plot in mice vaccinated with the plasmid constructs.

The effect on immunogenicity with respect to India specific HPV 16 L1 variations and validation of *in-silico* results was evaluated. Around 100 μg of prepared plasmid constructs of reference and variants i.e. pV8R & pV8V and pV16R & pV16V, respectively were injected in BALB/c mice quadriceps muscles at four weeks interval for three times. After two weeks of final injection, isolated serum of mice were proceeded for evaluation of anti-HPV-16 L1 antibody titer by ELISA. An induction of circulating IgG class anti-HPV16 L1 antibodies was observed in vaccinating mice (Supplementary Fig. S2). On comparison between the group injected with pV16V variant construct and the respective reference group, an elevated level of antibody titer (~2.7 folds, p < 0.002) was observed (Fig. 4). On the other hand, *in-vivo* results of pV8V variant construct showed a decrease in antibody titer (~0.4 folds, p < 0.393) in comparison to the reference construct (Fig. 4), which validated our *in-silico* results.

Together these results suggest that pV16V variant construct appears to be highly immunogenic with significantly higher level of antibody titer and it could be a promising candidate for the development of DNA vaccine against HPV.

## Discussion

In India, CaCx is the most common cancer among females with an annual incidence of about 134,000 cases^21^,^22^. Persistent infection with HR-HPVs is associated with precancerous lesions with the ultimate development of CaCx and HPV 16 infection is highly prevalent in India^23^. Intratypic sequence variation in HPV gene found in a particular geographical region can be of functional significance and may confirm the different oncogenic potentials^24^. It has been reported from various studies that gene variant T350G of HPV-16 was found to display more efficient degradation of Bax and strong binding to E6 binding protein. The alteration in amino acid at this position can alter the protein properties which may be important for its carcinogenic potential^25^. It is also showed that the variation T350G in HPV-16 E6 gene imparted an approximately 2 fold higher risk of viral persistence than prototype^26^,^27^. So, it is imperative to understand the geographical variant of the HPV for the better impact of the aimed vaccines development against it.

In the present study, around 92.0% cases showed positivity for HPV, which are in accordance with the previous studies^7^,^9^,^28^. The high prevalence of HPV-16 (95.6%) in the present study is also fairly similar to the other studies^7^,^8^ and is the most prevalent HPV type in India. The very low prevalence (1.7%) of HPV-18 type was not in accordance with the reports from the other parts of the world^28^,^29^,^30^.

The variation in HPV-16 L1 may affect the conformation-dependent L1 epitopes that are significant for virus neutralization^15^. The variation V8 seems to be important as a polar uncharged amino acid is replaced by a non-polar amino acid, which may play an important role in the immune recognition by the host system^16^,^17^,^18^. Three variations (V3, V12 and V13) observed in 100% of the samples, were absent in two studies from India^16^,^18^, but reported by Pillai *et al.*,^17^. Variation H202D (V3) has been shown to be responsible for viral assembly^10^,^31^,^32^. The other variations at C6163A, G6171A, T6245C, A6314G, A6432G, C6557T, G6719A, C6852T, C6863T, C6968T G6692A and G7058A/T have also been observed in other studies from India^16^,^17^. There was no correlation between the distributions of specific HPV-16 viral variants with the tumor stage. These results are in accordance to a study from Argentina which did not found any correlation of their variants with different tumor staging^33^.

The development of the neutralizing antibody against major capsid protein (self assembles) to form VLP of HPV shows a lot of promise for the prevention of papillomavirus-associated cancer and two available VLP based vaccines Gardasil and Cervarix currently in use. However, their high cost of production and ineffectiveness against other strains of HR-HPV as well as not having therapeutic value^34^,^35^,^36^ limits their use in low resource countries. There may be a possibility that intratypic HPV variant restrict the immune response by escaping consensus B-and T-cell epitopes of the available vaccines. These variants may also provide some new epitopes for targeting a particular geographical population, which may not be presented by these available vaccines. The prediction of B-and T-cell epitopes by bioinformatics tools may helpful in the vaccine development by reducing the experimental cost^37^.

It was reported that hypervariable epitope constructs (HEC)-immunized mice showed breath of reactivity and represent the immunodominant B-cell epitopes of the major capsid proteins of HPV^19^. This study showed the use of HECs for the development of vaccine against multiple strains of HPV. Plasmid construct of V16, present in HEC region, showed ~two fold higher antibody titer than the reference DNA vaccine construct which indicated that variant construct may be more immunogenic for the development of vaccines. For, variant construct (V8), B cell epitopes predicted by the ElliPro tool for reference sequence was ISTSETTYKNTN and had a score of 0.636 (with Thr having 0.833) whereas for variant sequence was ISTSEPTYKNT with a score of 0.630 (with Pro having 0.796). The overall predicted score of variant epitope (0.630) was less than predicted reference epitope (0.636), this may be due to the higher score of individual amino acid like 0.833 for Thr than Pro having 0.796 score. The overall score was measured as the mean of the individual amino acid score. On comparison of results, it can be interpreted that reference sequence is more antigenic than the variant sequence as the score of reference epitope was higher than the variant. Our *in-vivo* experimental results also confirmed that the variant construct had ~0.4 fold less antibody titer than reference DNA vaccine construct. Probably, this may be due to the structure constraint of proline having pyrrolidine ring which gives unusual shape resulting into conformational constraints or secondary structural preferences (Fig. 1). On the basis of these results, the present study showed the importance of *in-silico* analysis for effective vaccine development by reducing the non-immunogenic regions.

Recently, several reports showed that naked DNA vaccination with HPV-16 L1 in mice/or pig could produce increased level of serum IgG, anti-L1antibodies^38^,^39^. It was also reported earlier that plasmid DNA having HPV 16 L1 gene could induce a protective immune response in mice model^40^. We observed a significant increase in immune response to pV16 variant DNA construct (~2.7 folds higher antibody titer, p < 0.002), while pV8 variant construct showed low immune response (~0.4 folds decrease in antibody titer) but not attain statical significance (p < 0.393) in comparison to the respective reference construct, which is also demonstrated by *in-silico* results. So, this study shows promising results which appear to be useful for the development of India specific cost effective DNA based vaccines.

In conclusion, there is no study in Indian population that shows the effect of different variations of HR-HPV, and their effect on pathogenicity/antigencity. Our results showed the major variations like T379P of full length L1 gene which may play important role in the immunogenicity against HPV by affecting the binding affinity of immunogenic peptide (epitope). On the other hand, L500F (V16) was found to be more immunogenic than the reference DNA vaccine constructs. T379P (V8) variation causes the reduction of immunogenicity of epitope present in their vicinity. Therefore, these variations may be important for the oncogenic potential of HPV-16 bearing these mutations. So, we need to target potent epitopic sites to evoke the effective immune response. These results show a roadmap for the development of epitope based vaccine and for the generation of effective response, there is a need to study the more prevalent variants of HPV in specific geographical populations. In future, on the basis of this study priority may be given on the development of multi-epitope DNA vaccine, which may be a better alternative for the development of effective immune response against different variants. There was a study showing the effect of different variants using series of VLP prepared, showed the structural importance of it to induce effective immune response (IgG) and viral assembly for HPV mediated carcinogenesis^41^. The limitation of the study is that the effect of individual variants may be analyzed using L1 variants VLPs for effective antigen presentation^41^. However, both of the variations (V8 and V16) did not show much effect on the viral assembly as mentioned earlier^41^. Furthermore, enhancement of the immunogenicity of the particular DNA vaccine construct may be done by using appropriate adjuvant to evoke stronger immune response.

## Methods

### Sample Collection

A total of 250 CaCx tissue biopsy samples were collected from the Cancer Clinic of Department of Obstetrics and Gynecology, Lok Nayak Jai Prakash Hospital, New Delhi and control tissue from Department of Obstetrics and Gynecology, Safdarjung Hospital, New Delhi. The samples comprised of 102 biopsies of tumor stage I + II and 148 biopsies of stage (III + IV). Standard International Federation of Obstetrics and gynecology (FIGO) criteria was used to determine the tumor stage. All the tissue biopsies were collected in sterile sample collection vials (Greiner, USA) containing chilled phosphate-buffered saline (PBS) and stored at −80 °C in a deep freezer for further processing. The study was approved by the ethics committee of the institute **(ICPO-ICMR/IEC/2007/P-001)**. Written informed consent from all the patients was obtained and the study was carried out in accordance with the principles of Helsinki declaration.

### DNA extraction and HPV detection

Genomic DNA was extracted by standard method using Proteinase-K digestion followed by phenol/chloroform treatment as described earlier^42^. Initial HPV diagnosis was performed by amplification using consensus primers MY09 and MY11 for detection of HPV infection. Further, HPV genotyping was done by PCR using specific primers for high-risk (HPV-16/18) as described earlier^28^,^43^.

### Molecular Variant detection

The full length L1 gene of HPV-16 positive cases was amplified using forward primer 5′-AGCATCGATACCATGGCTCTTTGGCTGCCTAGTG AGG-3′ and reverse primer 5′-GCGGCCGCTTACAGCTTACGTTTTTGCGTTTAGCA GTTGTAG-3′for 1.5 kbp product. The amplified products were purified by Exonuclease-Shrimp Alkaline Phosphatase (Exo-SAP) treatment and directly sequenced according to the manufacturer’s protocol (ABI-3103^xl^). For variant analysis, Multiple Sequence alignment (MSA) of sequenced sample was done by Mega 5.1 software^44^ and compared with the reference sequence (K02718) for the major variant analysis (>15% of the samples).

### Sequence analysis and Epitope Prediction

Nucleotide sequences were translated by translate tool of ExPASy server (http://web.expasy.org/translate/) for determination of amino acid change. Computation of various physical and chemical parameters of protein sequences was calculated by Protparam, an online server (http://web.expasy.org/protparam/)^45^. For secondary structure prediction, PSIPRED server (http://bioinf.cs.ucl.ac.uk/psipred/) was used, which provides a simple and accurate secondary structure prediction method. Using a very stringent cross validation method to evaluate the method’s performance, PSIPRED3.2 achieves an average Q3 score of 81.6%^46^. Epitopes were predicted in reference and variant sequences using ElliPro of Immune Epitope Database (IEDB): analysis resource (http://www.iedb.org/).

### Structure Modeling

The structure of variant and reference sequence was modeled by taking HPV L1 structure (PDB: 1DZL A) as template with Modeller 9.10 of Sali lab^47^. The overall structural assessment of the modeled protein was checked using the SAVES Server (NIH) which contains PROCHECK, WHATCHECK and VERIFY3D programs for structural quality estimation. The selected model was visualized using UCSF Chimera, a structural visualization tool developed by the Resource for Bio-computing, Visualization and Informatics^48^. The predicted epitopes were docked using rigid-body docking methods, PatchDock algorithm (http://bioinfo3d.cs.tau.ac.il/PatchDock/index.html) and the refinement of the protein-protein docking solutions was done using FireDock (http://bioinfo3d.cs.tau.ac.il/FireDock/index.html) and the best model was selected on the basis of the lowest global energy.

### Construction of the HPV-16 L1 Recombinant Plasmid

The segment of L1 gene having V8 and V16 variants and their reference was amplified by using specific primers (Supplementary Fig. S3). The amplified products were cloned into CMV expression vector pcDNA3 (Invitrogen, CA), according to the manufacturers protocol. Plasmid constructs were extracted by standardized alkaline-lysis method and purified by EndoFree Plasmid Maxi Kit (Qiagen, Valencia, CA). The confirmation of correct orientation of the insert was done by sequencing.

### Genetic immunization

Plasmid DNA was dissolved in 0.9% saline without adjuvant at a concentration of 1 mg/ml. A total of 36 BALB/c mice (6 ± 8-week-old) were grouped into control, pcDNA, 16R, 16V, 8R and 8V (n = 6/group) and immunized intramuscularly with 50 μg of plasmid into each quadriceps muscles (total 100 μg), three times at 4 weeks interval. Blood was taken before each immunization and the mice were sacrificed two weeks after the third injection, serum was separated and stored at −80 °C. The animal study was approved by animal ethics committee at Dr. B. R. Ambedkar Center for Biomedical Research, University of Delhi, Delhi **(ACBR/12/IAEC/2856(a)).**

### Detection of Anti-HPV16-L1 Antibodies

The antibody titer in response to injected constructs has been detected by direct Enzyme-linked immunosorbent assay (ELISA) according to the method described previously^49^. In brief, ELISA plates were coated with HPV-16 VLP in cold PBS at a concentration of 1μg/mL and control plates with PBS by incubating at 4 °C overnight. After incubation, the plates were washed with wash-buffer PBS–0.5% Tween-20 (PBS-T) five times. Also, 10% horse serum in PBS (HS-PBS) was added to block the plates for 1 hour at room temperature (RT). Serum samples were diluted in HS-PBS (1:10,1:31.5,1:100) added to the plates and incubated for 2 hours at RT. Following five washes, anti-mouse IgG HRP (diluted 1:1000 in HS-PBS) was added to the plates and incubated for 1 hour at RT. After washing five times, peroxidase substrate ABTS was added and incubated for 30 min. Absorbance was measured immediately at 405 nm without addition of stop-solution.

### Statistical Analysis

All the statistical analysis was calculated using GraphPad InStat version3.0 software. One way ANOVA was used to determine the statistical significance of experimental groups with control. For statistical significance of reference and their respective variant were analyzed by applying Mann Whitney test (non parametric test). The p-values less than 0.05 were considered significant.

## Additional Information

**How to cite this article**: Kumar, A. *et al.* Identification and validation of immunogenic potential of India specific HPV-16 variant constructs: *In-silico* & *in-vivo* insight to vaccine development. *Sci. Rep.* **5**, 15751; doi: 10.1038/srep15751 (2015).

## Supplementary Material

Supplementary Information

## Figures and Tables

**Table 1:** Major variations of HPV-16 full length L1 sequence in cervical cancer.

S. No.	V1	V2	V3	V4	V5	V6	V7	V8	V9	V10	V11	V12	V13	V14	V15	V16	V16
Position	6163	6171	6240	6245	6314	6432	6557	6693	6719	6852	6863	6901	6590	6968	6992	7058	7058
Ref N	**C**	**G**	**C**	**T**	**A**	**A**	**C**	**A**	**G**	**C**	**C**	**—**	**GAT**	**C**	**G**	**G**	**G**
**A**	A	A	—	—	—	—	—	—	A	—	—	InsATC	DELETED	—	A	A	—
**T**	—	—	—	—	—	—	T	—	—	T	T			T	—	—	T
**G**	—	—	G	—	G	G	—	—	—	—	—			—	—	—	—
**C**	—	—	—	C	—	—	—	C	—	—	—			—	—	—	—
**% of Sample**	**24.13**	**17.24**	**100**	**24.13**	**24.13**	**96.55**	**24.13**	**24.13**	**24.13**	**34.48**	**24.13**	**100**	**100**	**24.13**	**24.13**	**10.34**	**24.13**
	202	205	228	—	—	292	—	379	—	—	435	448	465	—	—	—	500
Amino Acid	T—N	A—T	H—D	—	—	T-A	—	T-P	—	—	P-L	INS- S	DEL-D	—	—	—	L-F
	Polar-Polar	Non-Polar-Polar	Polar-Polar			Polar-Non-Polar		Polar-Non Polar			Non Polar-Non Polar	Polar	Polar				Non Polar-Non Polar
Hydrophobacity	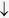	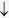	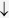			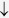		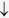			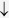						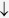
