# Supplementary material for: Identification and validation of immunogenic potential of India specific HPV-16 variant constructs: In-silico & in-vivo insight to vaccine development
Source: Sci Rep. 2015 Oct 28;5:15751. doi: 10.1038/srep15751 (PMC4623767; doi:10.1038/srep15751)
Supplement: Supplementary Information [file srep15751-s1.pdf]

Identification and validation of immunogenic potential of India specific HPV-16 variant constructs: *In-silico* & *in-vivo* insight to vaccine development

Anoop Kumar[1,3#,] Showket Hussain[1#,] Gagan Sharma[1], Ravi Mehrotra[2], Lutz Gissmann[3], Bhudev C Das[4,5*] and Mausumi Bharadwaj[1*]

[1]Division of Molecular Genetics & Biochemistry; [2]Division of Cytopathology Institute of Cytology & Preventive Oncology (ICMR), Noida, Uttar Pradesh, India

[3]Division of Genome Modification and Carcinogenesis, German Cancer Center, DKFZ Heidelberg, Germany.

[4]Dr. B.R. Ambedkar center for Biomedical Research, University of Delhi (North Campus), New Delhi, India.

[5]Present Address: Amity Institute of Molecular Medicine and Stem Cell Research (AIMMSCR) Amity University, Sector 125, Noida 201313, Uttar Pradesh, India.

[#] Authors contributed equally

***Correspondences**

Mausumi Bharadwaj, Scientist F
Division of Molecular Genetics & Biochemistry,
Institute of Cytology & Preventive Oncology (ICMR),
I-7, Sector 39, Noida 20130, India.
Tel: 91-95120-2579471, Fax: 91-95120-2579473
E-mail: mausumi.bharadwaj@gmail.com, bharadwajm@icmr.org.in

and,

Prof. Bhudev C Das
Amity Institute of Molecular Medicine and Stem Cell Research (AIMMSCR) Amity University, Sector 125, Noida 201313
Uttar Pradesh, India
E-mail: bcdas48@hotmail.com; bcdas@amity.edu

Supplementary Table S1: Amino acids composition in reference and variant full length L1 sequence of HPV-16

**S. No.**	**Amino Acid**	**Reference (%)**	**Variant (%)**	**S. No.**	**Amino Acid**	**Reference (%)**	**Variant (%)**
1	Ala	5.50%	5.50%	11	Leu	8.30%	8.30%
2	Arg	3.60%	3.60%	12	Lys	6.40%	6.40%
3	Asn	**5.60%**	**5.80%**	13	Met	2.10%	2.10%
4	Asp	5.30%	5.30%	14	Phe	**5.10%**	**5.30%**
5	Cys	2.40%	2.40%	15	Pro	7.00%	7.00%
6	Gln	4.00%	4.00%	16	Ser	**6.00%**	**6.20%**
7	Glu	4.00%	4.00%	17	Thr	**8.30%**	**7.90%**
8	Gly	6.60%	6.60%	18	Trp	1.30%	1.30%
9	His	**2.30%**	**2.10%**	19	Tyr	4.70%	4.70%
10	Ile	4.90%	4.90%	20	Val	6.80%	6.80%

Supplementary Figures

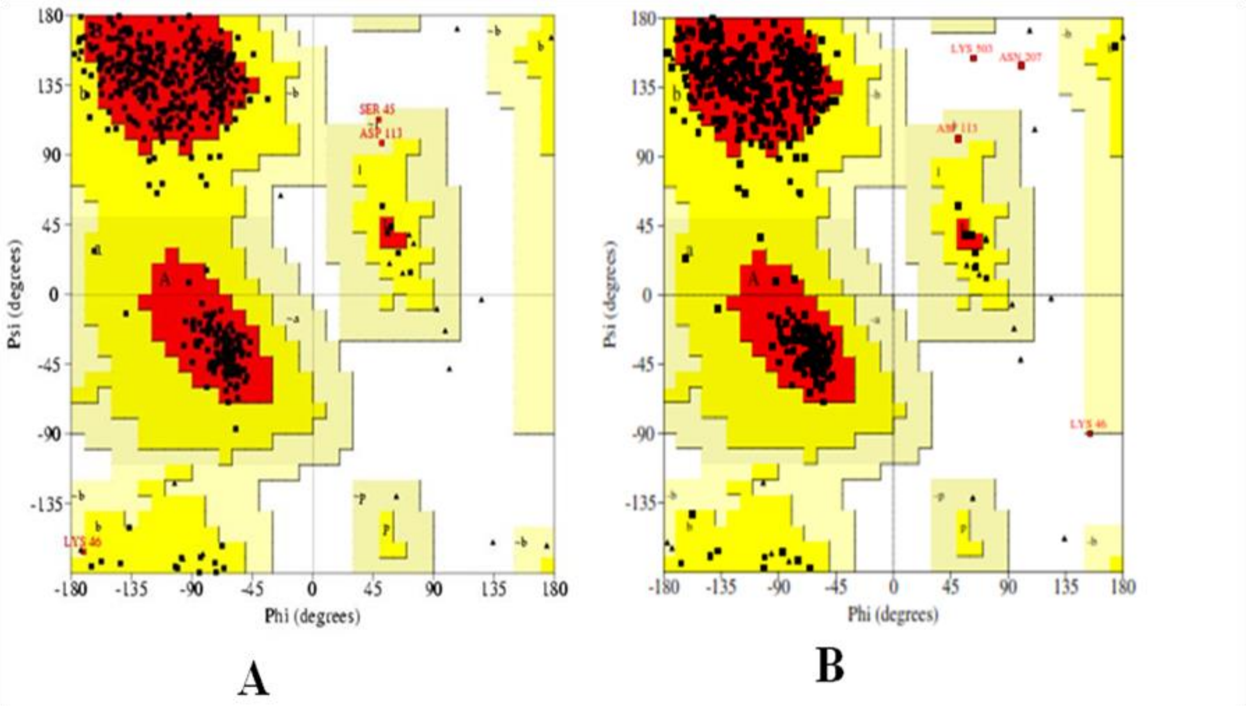

Supplementary Figure S1: Ramachandran plot of full length L1 (A) reference & (B) variant modeled protein. The most favoured region is color red, additional allowed region is color yellow, generously allowed colored light yellow and disallowed region shown in white field.

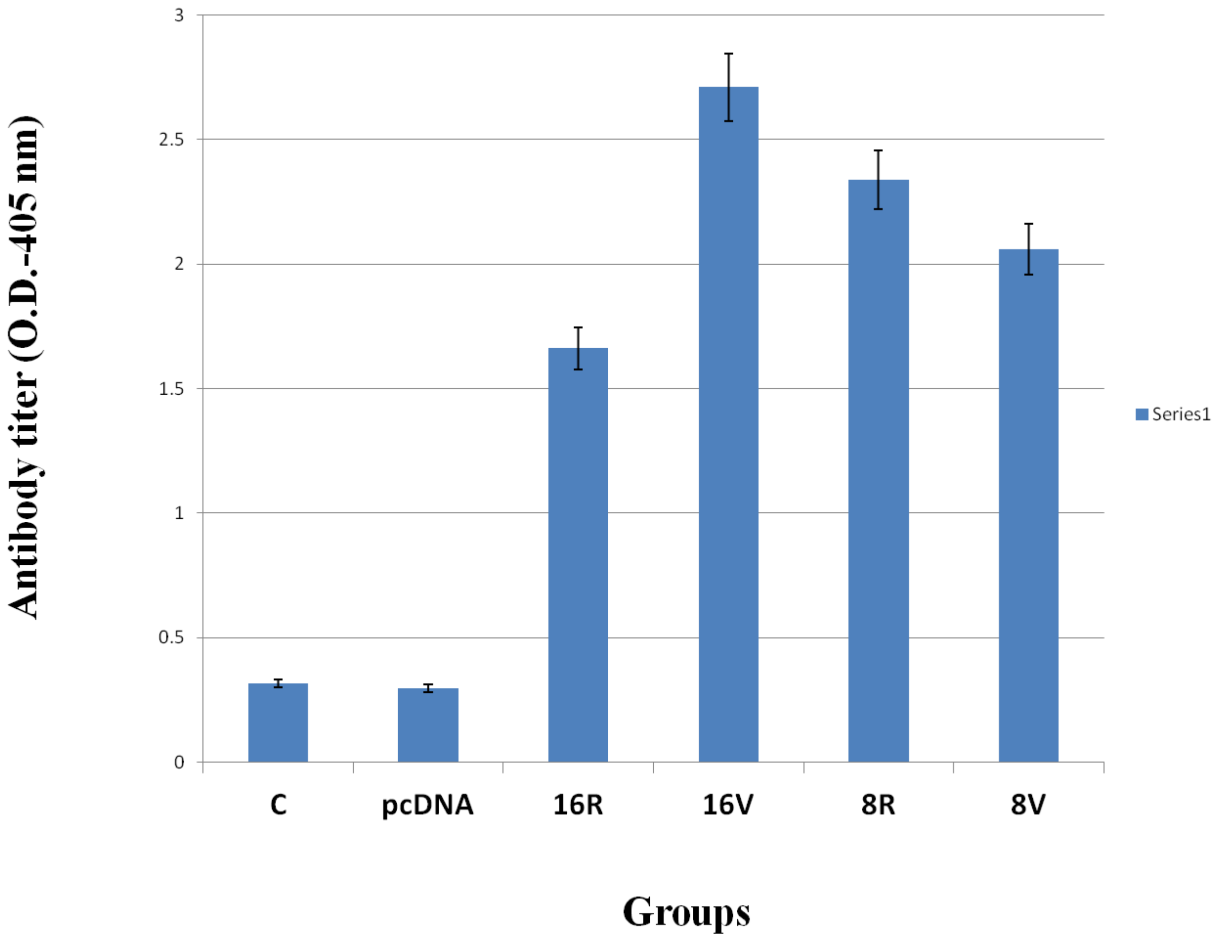

Supplementary Figure S2: Antibody titer of mice immunized with variant & their reference DNA vaccines construct of Control, pcDNA, 16R, 16V, 8R and 8V group.

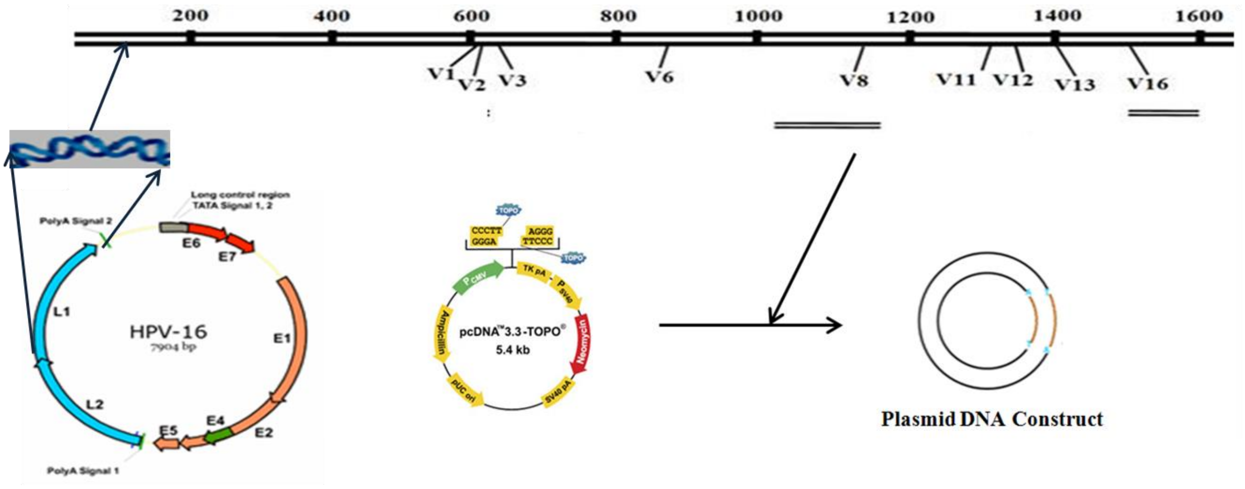

Supplementary Figure S3: Schematic representation of selection and cloning strategies for vaccine map
